# The role of the humoral immune response in the molecular evolution of the envelope C2, V3 and C3 regions in chronically HIV-2 infected patients

**DOI:** 10.1186/1742-4690-5-78

**Published:** 2008-09-08

**Authors:** Pedro Borrego, José Maria Marcelino, Cheila Rocha, Manuela Doroana, Francisco Antunes, Fernando Maltez, Perpétua Gomes, Carlos Novo, Helena Barroso, Nuno Taveira

**Affiliations:** 1 URIA-CPM, Faculdade de Farmácia de Lisboa, Avenida das Forças Armadas, 1649-019 Lisbon, Portugal; 2 UTPAM, Departamento de Biotecnologia, Instituto Nacional de Engenharia Tecnologia e Inovação, Estrada Paço Lumiar 22, 1649-038 Lisbon, Portugal; 3 Serviço de Doenças Infecciosas, Hospital de Santa Maria, Avenida Professor Egas Moniz, 1600-190 Lisbon, Portugal; 4 Serviço de Doenças Infecciosas, Hospital de Curry Cabral, Rua Beneficência 8, 1050 Lisbon, Portugal; 5 Laboratório de Biologia Molecular, Serviço de Medicina Transfusional, Centro Hospitalar Lisboa Ocidental, Hospital Egas Moniz, Rua Junqueira 126, 1349-019 Lisbon, Portugal; 6 Instituto Superior de Ciências da Saúde Egas Moniz, Quinta Granja, Campus Universitário, 2829-511 Caparica, Portugal

## Abstract

**Background:**

This study was designed to investigate, for the first time, the short-term molecular evolution of the HIV-2 C2, V3 and C3 envelope regions and its association with the immune response. Clonal sequences of the *env *C2V3C3 region were obtained from a cohort of eighteen HIV-2 chronically infected patients followed prospectively during 2–4 years. Genetic diversity, divergence, positive selection and glycosylation in the  C2V3C3 region were analysed as a function of the number of CD4+ T cells and the  anti-C2V3C3 IgG and IgA antibody reactivity

**Results:**

The mean intra-host nucleotide diversity was 2.1% (SD, 1.1%), increasing along the course of infection in most patients. Diversity at the amino acid level was significantly lower for the V3 region and higher for the C2 region. The average divergence rate was 0.014 substitutions/site/year, which is similar to that reported in chronic HIV-1 infection. The number and position of positively selected sites was highly variable, except for codons 267 and 270 in C2 that were under strong and persistent positive selection in most patients. N-glycosylation sites located in C2 and V3 were conserved in all patients along the course of infection. Intra-host variation of C2V3C3-specific IgG response over time was inversely associated with the variation in nucleotide and amino acid diversity of the C2V3C3 region. Variation of the C2V3C3-specific IgA response was inversely associated with variation in the number of N-glycosylation sites.

**Conclusion:**

The evolutionary dynamics of HIV-2 envelope during chronic aviremic infection is similar to HIV-1 implying that the virus should be actively replicating in cellular compartments. Convergent evolution of N-glycosylation in C2 and V3, and the limited diversification of V3, indicates that there are important functional constraints to the potential diversity of the HIV-2 envelope. C2V3C3-specific IgG antibodies are effective at reducing viral population size limiting the number of virus escape mutants. The C3 region seems to be a target for IgA antibodies and increasing N-linked glycosylation may prevent HIV-2 envelope recognition by these antibodies. Our results provide new insights into the biology of HIV-2 and its relation with the human host and may have important implications for vaccine design.

## Background

The etiologic agents of AIDS, HIV-1 and HIV-2, are two distinct human lentiviruses with similar structural and genomic organization but sharing only 50% of genetic similarity [1]. Compared to HIV-1, the infection by HIV-2 is associated with better prognosis, slower disease progression and transmission, longer latency period and reduced mortality rate [2-6]. Moreover, most HIV-2 patients have normal CD4^+ ^T cell counts and low or undetectable plasmatic viral levels [7,8]. Two possible explanations for these differences may be the slower replication capacity of HIV-2 and a more efficient immune control of HIV-2 [9-13].

The *env *gene codes for the viral envelope glycoproteins, which are responsible for HIV entry into cells [14]. Rapid evolutionary changes and high genetic variability are two major characteristics of the HIV *env *gene [15]. In HIV-1 infection, conflicting associations have been reported between disease status and within-patient *env *gene evolution. Hence, some studies have shown that genetic diversity and divergence from the infecting strain increase during HIV-1 infection but become stable or even decrease in the advanced stage of disease, with the lower CD4^+ ^T cell counts and progression to AIDS [16-18]. Other authors have shown that higher genetic diversity and divergence are found in patients with rapid progression to disease than in slow- or non-progressors [19,20]. There is also a positive correlation between viral replication and intrahost HIV-1 evolution in elite controllers and long-term nonprogressors [21].

The number of studies investigating within-patient HIV-2 molecular evolution and their association with clinical and immunological evolution is limited. In one transversal study, we have shown that the genetic diversity of the HIV-2 *env *may be directly related to the period of infection [22]. Longitudinal studies performed in Senegal have shown that higher variability in the *env *V3 region is generally found in patients with faster disease progression to AIDS [23] and that in elite controllers (patients infected for ≈ 10 years with normal CD4^+ ^T cell counts without antiretroviral therapy and with low or undetectable viral load) the rate of *env *gene diversification may be positively associated with the rate of CD4^+ ^T cell number decrease [24].

Higher rate of molecular evolution, with predominance of nonsynonymous amino acid substitutions, tends to occur in regions of the HIV-1 *env *gene submitted to strong selective pressure from the immune system [15,25-28]. A structure of particular importance in this process is the V3 loop of the surface glycoprotein which is essential for HIV coreceptor usage [29-32] and for inducing the production of neutralizing and nonneutralizing antibodies in HIV infected individuals [33]. Neutralizing antibody responses, both autologous [34-36] and heterologous [36,37] may be more common in HIV-2 than in HIV-1 infection. Still, little is known about the role of humoral immunity in the evolution of the HIV-2 *env *gene. In the present study we analyze, for the first time, the molecular evolution of the *env *C2V3C3 regions in chronically HIV-2 infected patients over a two to four year period in the context of their antibody response (IgG and IgA) against the same envelope region.

## Methods

### Patients

Eighteen HIV-2 patients attending different hospitals in Lisbon, Portugal, were followed prospectively during 2–4 years (Table 1). Fourteen patients were taking reverse transcriptase and/or protease inhibitors. During the follow-up period three patients (PTHCC20, PTHSM9 and PTHSM10) had detectable plasma viral load. Eight patients had < 200 CD4^+ ^T cells/μl (AIDS defining condition).

**Table 1:** Virological and immunological characterization of the patients

Patient	Year of diagnosis	Sample	CD4^+ ^T cells/μl	RNA copies/ml	Antiretroviral therapy	Antibody reactivity against C2V3C3 (OD/cut-off)	
						IgG	IgA
PTHCC1	2001	2003	308	<200	+	14.4	1.41
		2005	319	na		13.3	1.49
PTHCC2	2003	2003	358	<200	+	24.3	1.69
PTHCC4	2000	2003	240	<200	+	9.6	3.26
PTHCC5	1993	2003	480	<200	+	20.1	2.40
		2004	na	<200		22.4	2.14
PTHCC7	2002	2003	144	<200	+	22.9	1.93
		2005	43	<200		22.1	2.42
PTHCC8	2000	2003	141	<200	+	19.4	3.98
		2005	350	na		17.1	3.69
PTHCC12	1995	2003	66	<200	-	28.0	5.79
		2004	84	na		25.5	2.67
PTHCC13	2004	2005	954	<200	-	na	na
PTHCC14	1998	2003	184	<200	+	23.6	3.82
PTHCC17	1998	2003	367	<200	+	22.5	2.74
		2004	270	<200		18.5	2.66
PTHCC19	2003	2003	175	na	+	26.5	1.82
		2004	400	<200		22.3	2.22
		2005	60	na		18.7	2.01
PTHCC20	1998	2003	78	na	+	24.5	1.57
		2004	73	5246		20.0	1.66
		2005	85	<200		19.9	1.37
PTHSM2	2002	2003	275	<200	+	5.9	1.55
		2004	65	<200		6.2	1.57
		2005	122	<200		10.9	1.93
		2006	172	<200		4.7	2.09
PTHSM3	1993	2005	1452	<200	-	7.7	3.47
PTHSM6	2001	2005	471	<200	+	13.9	5.24
PTHSM7	1996	2003	587	na	-	11.4	2.39
PTHSM9	1996	2003	15	<200	+	neg	0.82
		2004	na	484		neg	0.79
PTHSM10	2001	2003	342	5804	+	neg	3.56
		2004	265	4792		neg	3.54
		2005	212	na		neg	3.79

na, not available; neg, no reactivity; +, yes; -, no.

### Quantification of HIV-2 plasma viremia

HIV-2 viremia in the plasma was quantified with a quantitative-competitive RT-PCR assay as described elsewhere [38].

### DNA extraction, PCR amplification, cloning and sequencing

PBMCs from all patients were co-cultivated with normal PBMCs to try to isolate virus [39]. At the end of the culture period, which is when the culture was positive (mean, 15 days), cells were harvested and DNA was extracted with the Wizard® Genomic DNA Purification kit (Promega) for subsequent analysis. A fragment of the C2V3C3 region (378 bp) of the HIV-2 *env *gene was amplified in a *nested *Polymerase Chain Reaction (PCR) as described previously [22]. PCR fragments were cloned into pCR^®^4-TOPO^® ^vector (Invitrogen) and transformed into One Shot^® ^Match1™-T1^R ^competent cells (Invitrogen). Cloned plasmids were extracted [40], purified and sequenced using BigDye Terminator Cycle sequencing kit (Applied Biosystems), with M13 Forward and Reverse primers, and an automated sequencer (ABI Prism 3100, Applied Biosystems). For each patient an average of 13 clones (range 7–21) was sequenced per sampling year.

### Sequence analysis and phylogenetic studies

The nucleotide sequences were aligned using Clustal X [41] and manual adjustments were made using Genedoc [42]. Genetic distances between sequences were calculated using the maximum composite likelihood method implemented in the MEGA version 4 [43]. Inter- and intra-sample synonymous (dS) and nonsynonymous (dN) distances were estimated using the modified Nei-Gojobory method with the Jukes-Cantor correction, also implemented in the MEGA software package.

Maximum likelihood analyses [44] were performed using the best-fit model of molecular evolution estimated by Modeltest under the Akaike information criterion [45]. The chosen model was TVM+G+I. Tree searches were conducted in PAUP version 4.0 using the nearest-neighbor interchange (NNI) and tree bisection and reconnection (TBR) heuristic search strategies [46], and bootstrap resampling [47]. The nucleotide divergence rate was estimated using an adaptation of the methodology previously described by Salazar-Gonzalez et al. [48]. Firstly, maximum likelihood trees were constructed for each patient using all clonal sequences from each time point and rooted with the consensus sequences from other patients. Then, assuming a molecular clock, the branch lengths between the leafs and the root of the tree were calculated by using Branchlength Calculator [49] and plotted against time in years.

Natural selection of specific amino acids was examined using Codeml, models M0 and M3, with the HYPHY package [50]. Potential N-glycosylation sites were identified using N-Glycosite [51]. The entropy at each position in protein alignment was measured with Shannon Entropy [52].

### Humoral antibody response against the env C2V3C3 regions

IgG and IgA antibody response against the *env *C2V3C3 region was quantified with the ELISA-HIV2 test developed in our laboratory, as described elsewhere with some modifications [53]. Briefly, microtiter plates (96-well) were coated with rgp36 and rpC2-C3 by overnight incubation at 4°C and blocked with 1% gelatine in Tris-buffered saline (TBS). HIV-2-positive plasma samples were added to the antigen coated wells at a 1:100 dilution. Bound antibodies were detected by using alkaline phosphatase (AP)-conjugated goat anti-human IgG (diluted 1:2000 in TBS) or horseradish peroxidase (HPR)-conjugated rabbit anti-human IgA (diluted 1:2000 in phosphate-buffer saline) (Sigma-Aldrich). The colour was developed using p-nitrophenilphosphate (p-NPP Tablets, Sigma-Aldrich) as chromogenic substrate to AP and *o*-phenylenediamine dihydrochloride (OPD) to HPR. Optical density (OD) was measured with an automated microplate reader LP 400 (Bio-Rad) at 405 and 492 nm against a reference wavelength of 620 nm. The clinical cut-off value of the assay, calculated as the mean OD value of HIV-seronegative samples plus three times the standard deviation [SD], was determined using samples from healthy HIV-seronegative subjects. The results of the assay are expressed quantitatively as OD_clinical sample(S)_/OD_cut-off(CO) _ratios. For ratio values >1 the sample is considered as seroreactive.

### Statistical analysis

Statistical analysis was performed in GraphPad Prism version 4.00 for Windows (GraphPad Software), with a level of significance of 5%. For the inter-patient statistical analysis across time, only information obtained from one time point (one sample) per patient was considered in order to guarantee the independence of the data analyzed. Thus, to maximize the number of observations in the analysis, we chose the first sample (first time point) available for each patient. Nonparametric tests were used to compare means and medians between variables: paired data was analyzed with Wilcoxon-matched pairs test and Friedman test; unpaired variables were tested with Mann Whitney U test and Kruskal-Wallis test. To study how two variables varied together linear regression was performed and Spearman correlation coefficients were computed. Finally, Deming linear regression was used to study the overall variation (slopes) of intra-patient data with time (longitudinal analysis).

### GenBank accession numbers

Sequences have been assigned the following GenBank accession numbers: EU358115–EU358499, EU358501, EU358504, EU358507, EU358509, EU358513, EU358517, EU358519–EU358521, EU358524, EU358525, EU358527–EU358531, EU358533, EU358536–EU358538, EU358541, EU358543, EU358546–EU358549, EU358551–EU358567, EU360797–EU360799.

## Results

### Phylogenetic relationships, genetic diversity and divergence

**Figure 1:**
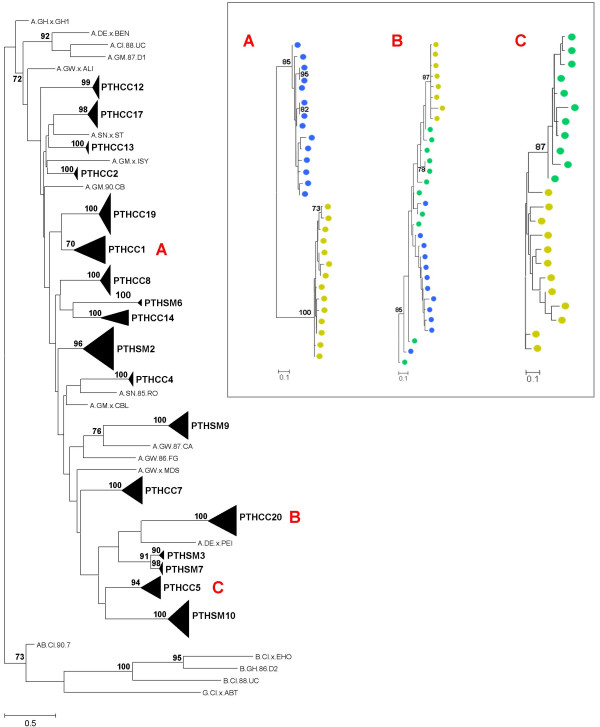
**Maximum-likelihood phylogenetic analysis**. The phylogenetic tree was constructed with reference sequences from HIV-2 groups A, B and G, under the TVM+G+I evolutionary model, using the NNI heuristic search strategy and 1000 bootstrap replications. The triangles represent the compressed subtrees containing clonal sequences obtained from all samples collected for each patient. The length of the triangle represents the intra-patient nucleotide diversity and its thickness is proportional to the number of sequences. The bootstrap values supporting the internal branches are shown. The scale bar represents evolutionary distances in substitutions per site. The inset contains the subtrees of patient PTHCC1 (A), PTHCC20 (B) and PTHCC5 (C) (Yellow circle – 2003; green circle – 2004; blue circle – 2005).

To investigate the molecular evolution of the HIV-2 *env *gene we have amplified, cloned and sequenced the *env *gene fragment coding for the C2, V3 and C3 regions using yearly samples collected from 18 patients followed prospectively for 2–4 years. A total of 431 clonal sequences were obtained from 18 patients (average of 13 sequences per patient per sampling year). Phylogenetic analysis showed that all sequences clustered together within HIV-2 group A and that each patient sequences formed monophyletic sub-clusters with high bootstrap supporting values (Figure 1). Phylogenetic analysis also showed that with the exceptions of patients PTHCC1, PTHCC5 and PTHCC20, sequences from most patients were not segregated according to sampling years, a clear indication that there were no major shifts in virus population structure from one year to the other.

The mean evolutionary distance between different nucleotide sequences from each sample/year (nucleotide diversity) was 2.1% (standard deviation = 1.1) (additional file 1). Nucleotide diversity was neither associated with clinical status (2.1% mean median genetic distance in AIDS patients *vs *1.4% in the other patients; *p *= 0.203) nor with plasma viremia (2.3% in viremic patients *vs *1.8% in aviremic patients; *p *= 0.386) (n = 18).

Considering the first and the last samples of each patient, nucleotide diversity increased along the course of infection in all patients, except for patient PTHCC5 (additional file 1). Shannon's entropy was used to measure the relative amino acid variability in our set of sequences [52]. The sum of entropy values of the amino acid alignments varied between regions (*p *< 0.001), being significantly lower for the V3 region (*p *< 0.001) and higher for the C2 region (*p *< 0.005) (additional file 1).

Within-patient nucleotide divergence rate was on average 0.014 substitutions per site per year for the C2V3C3 region, but it varied widely between patients (SD = 0.011). There was no association between the divergence rate and the variation in the number of CD4^+ ^T cells over time (Deming regression analysis, F = 0.058, *p *= 0.816). Likewise, the divergence rate of the C2V3C3 regions was not related with the level of IgG antibodies produced against the homologous peptide over time (F = 0.192, *p *= 0.675).

### Selection analysis and adaptation rate of the C2, V3, and C3 regions

**Figure 2:**
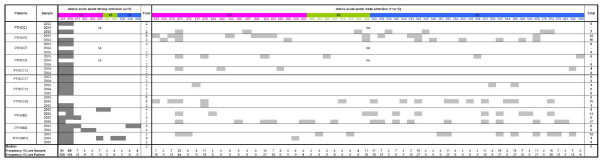
**Frequency, intensity and distribution of positively selected sites in the C2, V3 and C3 regions along the course of HIV-2 infection**. Positively selected codons (obtained with Codeml, model M3) were classified in two categories according to the ω ratio:ω>6, codons under strong selective pressure; 1<ω<6, codons under weak selective pressure. The frequency and distribution of positively selected sites in the C2, V3 and C3 regions are shown in each infection year. Higher frequency positively selected sites are shown in bold letters. Sites were numbered according to the reference HIV-2ALI strain. (na, not available)

Intra-patient analysis showed that the overall C2V3C3 region was under purifying selection (dN/dS ratio < 1) along the course of infection in all patients (additional file 1). Analysis of the number and location of positively selected codons is useful to identify particular amino acids that may be under the selective pressure of the immune system, regions that can define potential neutralizing epitopes or that are functionally important for the protein [15,25-28]. In the present study, higher number of sites under positive selection tended to be found in patients with detectable viremia compared to patients with undetectable viremia (median, 15 sites *vs *2; *p *= 0.061) (n = 18) (additional file 1). Otherwise, the number of positively selected sites was highly variable in number and position in most patients (Figure 2). Notable exceptions were amino acids at positions 267 and 270 in C2 (numbered according to the reference HIV-2ALI strain) that were under strong positive selection in all patients. Selection at these two sites persisted for at least two years in 9 patients (Figure 2). Because of these two sites, the median number of positively selected codons per sample was higher in the C2 region compared with the other regions (*p *< 0.005) (n = 18). Finally, using linear regression analysis we found that within each patient an average of 1.0 (SD = 3.8) positively selected site varied per year (adaptation rate).

### Glycosylation of the HIV-2 env C2-C3 region

**Figure 3:**
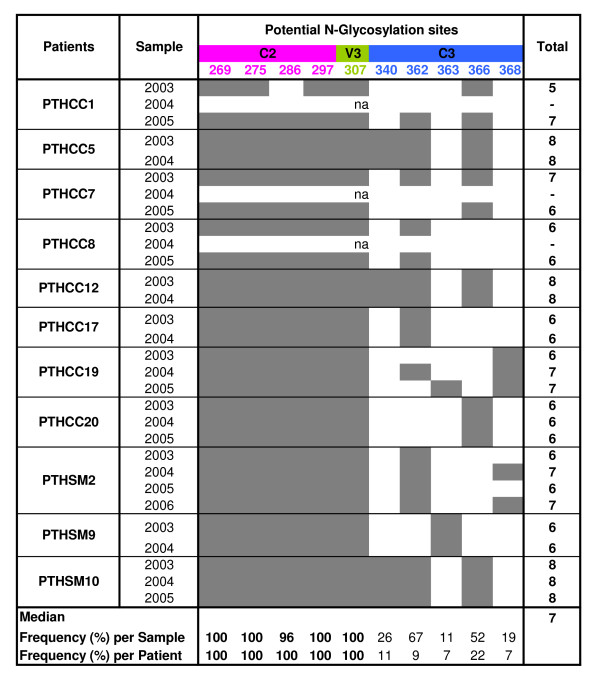
**Frequency and distribution of potential N-glycosylation sites in the C2, V3 and C3 regions along the course of infection**. The frequency and distribution of potential N-linked glycosylation sites in the C2, V3 and C3 regions are shown in each infection year. Higher frequency glycosylation sites are shown in bold letters. Sites were numbered according to the reference HIV-2ALI strain. (na, not available)

Since the glycosylation pattern of the HIV-1 *env *gene may influence neutralization escape to the immune system, viral tropism and clinical progression [32,36,54-57], we determined the number of potential N-glycosylation sites in our sequences and examined its variation as a function of time and other parameters analyzed in this study. The number of N-glycosylation sites ranged from 5 to 8 (median, 7) and tended to be conserved along the infection in each patient, the exception being patient PTHCC1 with an increase in two sites over the three years of follow up (Figure 3). The number of glycosylation sites varied significantly between C2, V3 and C3 (*p *< 0.001), being concentrated particularly in C2 (*p *< 0.001) (n = 18). At the intra- and inter-patient level, the most conserved N-glycosylation sites were located in C2 and V3. With one exception, all sites that varied over time were located in C3. The number of N-linked glycosylation sites was directly associated with the number of positively selected sites (r^2 ^= 0.301; *p *= 0.018).

### Molecular evolution of the C2, V3 and C3 regions as a function of the antibody response

**Figure 4:**
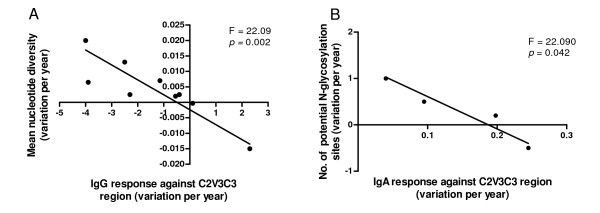
**C2V3C3 sequence evolution along the course of infection as a function of antibody response**. Deming regression analysis. (A) Annual variation (slope) of the C2V3C3-IgG response vs Annual variation (slope) of the mean nucleotide diversity; (B) Annual variation (slope) of the C2V3C3-IgA response vs Annual variation (slope) of the number of potential N-glycosylation sites.

All patients produced IgA antibodies against the C2V3C3 region whereas IgG antibodies were detected in all but two patients, PTHSM9 and PTHSM10 (Table 1). Intra-patient analysis revealed that along the course of the infection the variation of C2V3C3-specific IgG response was inversely associated with the variation of nucleotide diversity (F = 22.09; *p *= 0.002) as well as with the dN rate (F = 22.800; *p *= 0.002) and amino acid diversity (Shannon's entropy, F = 23.610; *p *= 0.002), particularly in the V3 (F = 11.660; *p *= 0.014) and C3 regions (F = 6.214; *p *= 0.041) (n = 9) (Figure 4). Variation of the C2V3C3- specific IgA response over time was inversely associated with variation in the number of N-linked glycosylation sites (F = 22.090; *p *= 0.042; n = 4) which occurred in four patients particularly in the C3 region (Figure 4).

## Discussion

In this study we have examined, for the first time, the molecular evolution of the envelope C2, V3 and C3 regions during chronic HIV-2 infection and its correlation with the antibody response against the same regions. Our cohort was constituted by long-term infected patients showing, in general, low CD4^+ ^T cell counts and undetectable plasma viremia.

Nucleotide diversity increased with time in all but one patient with values similar to those obtained in an earlier study performed with HIV-2 elite controllers (2.1%, this study, vs 1.7%; *p *= 0.3440) [24]. This value is also similar to the 2.5% median diversity reported for chronically HIV-1 infected patients [58] and to the 3.0% mean diversity reported for some long-term nonprogressors with low viral load [21].

In phylogenetic analysis we found low quasispecies complexity in most patients, i.e. virus populations from most patients were mostly homogeneous during the follow up period. This was expected since HIV-2 is generally seen as a slowly evolving virus and over a short period of time one would expect to observe few evolutionary changes [22,24,59]. However, in three patients there was evidence for segregation of virus quasispecies according to the year of infection, which implies high rate of evolutionary change and immune selection in these patients [15,60]. Consistent with this, we found that the nucleotide divergence rate varied widely between patients. Moreover, the average nucleotide divergence rate (0.014 substitutions per site per year) was very high when compared to that reported for HIV-2 elite controllers (mean, 0.23%) [24] and for HIV-1 long-term non progressors with low plasma viral load (mean, 0.27%) [21]. Even though we could not detect any association between nucleotide divergence and the number of CD4^+ ^T cells, the higher net divergence observed in our patients might be related to their high immune deterioration, as higher genetic divergence is generally found in HIV-1 rapid progressors compared to slow- or non-progressors [19,20]. In fact, the 0.014 annual divergence rate found in our patients is similar to that found in chronically HIV-1 infected patients (between 1.0% and 1.5% per year) [17,58]. In conclusion, the sampling schedule used in our study, and possibly the fact that we have analyzed the virus present inside the cells and not in the plasma, has enabled us to demonstrate that the evolutionary dynamics of HIV-2 during chronic infection is surprisingly similar to HIV-1. This implies that HIV-2 is actively replicating during chronic infection, possibly in the lymphoid tissue, as in HIV-2 patients the mononuclear cells in the lymph nodes are heavily infected, even more than the mononuclear cells in the peripheral blood [61,62]. Future studies of HIV-2 nucleotide divergence should include also the virus populations present in the lymphoid tissue and other cellular compartments (e.g. GI tract).

Despite the high nucleotide divergence rate, most of the substitutions were of a synonymous nature such that the dN/dS ratio of the C2V3C3 region was always below one and, most importantly, it decreased over time in most patients. These results are in agreement with previous reports that have examined the C2V3C3 region [22,24] and with the observation that, globally, the HIV-2 *env *gene is under purifying selection [25]. Consistent with previous studies of a cross-sectional nature, we found that C2 and C3, but not V3, were the fastest evolving regions at the nucleotide and amino acid level contributing significantly to the high within-patient nucleotide divergence rate [22,63]. The conservation of the V3 region *in vivo *implies that in HIV-2, as in HIV-1, this region is submitted to strong structural and conformational constraints which are probably related to its crucial functional roles at the level of coreceptor binding and cell entry [29-32].

It is probable that adaptation to immune pressure is the main driver of the rapid intra-host evolution of the C2 and C3 regions in HIV-2 [15,25,58,60,64-66]. Indeed, we found that most of the amino acids under selection are located in C2, including the two amino acids that are under strongest positive selection in all patients (positions 267 and 270). Moreover, selection at these two sites persisted for at least two years in the majority of the patients which is a clear indication that they are under continued immune pressure *in vivo *[60,67]. The equivalent amino acids in HIV-1 are not under positive selection [67], are located in the hidden surface of envelope glycoprotein complex [58] and define a cytotoxic T cell epitope [68]. Thus, our results also suggest that the antigenic presentation of the C2, and perhaps the C3 region (see below), in the envelope complex of HIV-2 differs substantially from that of HIV-1.

Glycans on HIV-1 envelope protein play an important role in the folding of the glycoproteins, in infection and in evasion from the host immune response (reviewed in [69]). We found that, as for HIV-1 [51,58], the majority of potential N-glycosylation sites were concentrated in the C2 region. The four N-glycosylation sites in C2 and the site in the beginning of V3 were highly conserved in all patients throughout infection which is strongly indicative of convergent evolution at these glycosylation hotspots and suggests an unexpected constraint on the potential diversity of the HIV-2 envelope [70,71]. The convergent evolution of glycosylation sites may have important implications for both vaccine design and antiviral therapeutic [69].

To try to identify the immune correlates of the molecular evolution of HIV-2 C2, V3 and C3 regions we have looked into all possible associations between the number of CD4^+ ^T cells or the IgA and IgG antibody levels and different parameters that reflect viral molecular evolution. In longitudinal analysis there was no significant association between the number of CD4^+ ^T cells and nucleotide diversity, amino acid entropy, nucleotide divergence, dN/dS ratio and number of positively selected sites. These results are in partial contrast to those of MacNeil et al. [24], who found a direct association between the rates of HIV-2 diversification and rates of CD4^+ ^T cell decline in long-term non progressors followed for a decade in Senegal. The short term follow-up and the associated modest variation in the number of CD4 ^+ ^T cells might have prevented the detection of this type of association in our patients.

Strikingly, however, there was a close relationship between virus diversification and evolution and C2V3C3-specific antibody response over time. In fact, higher IgG response was significantly associated with lower viral variability at the nucleotide and amino acid levels as well as with lower frequency of nonsynonymous substitutions. These results imply that the anti-C2V3C3 IgG antibodies are effective at reducing viral population size limiting the number of virus escape mutants [72]. This is in striking contrast to the majority of acute and chronic HIV-1 infections where the virus quickly escapes from anti-V3 and anti-C3 autologous neutralizing antibodies [33,73-76]. Consistent with the lower capacity of HIV-2 to escape from C2V3C3- neutralizing antibodies when compared to HIV-1, we found that on average HIV-2 has a five-fold lower adaptation rate *in vivo *than HIV-1 (1 positively selected site per year vs 5 sites per year) [60,77]. The HIV-2 low adaptation rate may be related to its low replicative capacity and low plasma viral load [12,13,78]. Overall, these results provide support for a crucial role of neutralizing antibody response in the effective containment of viral replication in HIV-2 infection *in vivo *[36].

Surprisingly, in some patients addition of glycans to the C3 region was associated with a reduction in the IgA immunogenicity of the C2V3C3 region. Envelope-specific plasma IgA antibodies, mostly binding to the gp36 transmembrane glycoprotein, have been found to neutralize HIV-2 [79]. Increasing the number of N-glycans in the envelope gp120 surface glycoprotein, or varying the position of glycosylation sites, has been associated with escape from IgG neutralizing antibody response in simian immunodeficiency virus (SIV) and HIV-1 infection [57,80-82]. Hence, one plausible explanation for the inverse association between IgA response and N-glycosylation is that the C3 envelope region induces IgA neutralizing antibodies to which HIV-2 escapes through the occlusion of the C3 region with N-linked glycans. This may have important implications for vaccine design. Ongoing studies will determine whether C2V3C3- specific IgA antibodies present in these patients effectively neutralize their autologous virus.

## Conclusion

The evolutionary dynamics of HIV-2 envelope during chronic and highly suppressed infection is surprisingly similar to HIV-1 implying that the virus is actively replicating in cellular compartments. Convergent evolution of N-glycosylation in C2 and V3, as well as the limited diversification of V3, indicates however that there are important functional constraints to the potential diversity of the HIV-2 envelope. HIV-2 envelope diversification is inversely related to the C2V3C3-specific IgG antibody response over time implying that these antibodies are effective at reducing viral population size, limiting the number of virus escape mutants. The C3 region seems to be a target for IgA antibodies and increasing N-linked glycosylation may prevent HIV-2 envelope recognition by these antibodies. Our results provide new insights into the biology of HIV-2 and its relation with the human host and may have important implications for vaccine design.

## Competing interests

The authors declare that they have no competing interests.

## Authors' contributions

NT designed and coordinated the study. PB performed most of the cloning and sequencing experiments. JMM isolated the viruses and quantified the antibody responses. HB and CR participated in virus isolation and in the sequencing analysis of some patients. MD, FA and FM recruited the patients and were responsible for collecting the blood samples and the clinical data. PG quantified the plasma viremia. CN and PG helped with the interpretation of data and revision of the manuscript. PB and NT preformed statistical analysis. PB and NT interpreted the data and wrote the manuscript. All authors reviewed and accepted the final manuscript.

## Supplementary Material

Additional file 1 Table 2. Results from sequence and phylogenetic analysis. ^a ^dN/dS – ratio of nonsynonymous and synonymous substitutions, obtained with Codeml (model M0). ^b ^dN/dS – ratio of nonsynonymous and synonymous substitutions between the first and the last time point, obtained with Codeml (model M0), when applicable. ^c ^Sum of Shannon's entropy values at each position in protein alignment. ^d ^Number of positively selected codons in the nucleotide alignment, obtained with Codeml (model M3). SD – Standard deviation. Click here for file
